# Strangulated Bochdalek Hernia in Adults: Timely Recognition and Surgical Intervention Can Prevent a Lethal Outcome

**DOI:** 10.7759/cureus.49420

**Published:** 2023-11-26

**Authors:** Siddharth S Das, Zaid AbdelAziz, Suhasini Krishnan, Feras H Alkhatib

**Affiliations:** 1 General Surgery, Dubai Hospital, Dubai, ARE; 2 Medicine, Dubai Academic Health Corporation, Dubai, ARE

**Keywords:** diaphragmatic hernia, resection, gangrenous, strangulated, adult bochdalek hernia

## Abstract

Bochdalek hernias are rare diaphragmatic hernias most commonly seen in pediatric populations. Adults with this condition may be asymptomatic or present with gastrointestinal symptoms such as abdominal pain, pressure, choking, or dysphagia. Computed tomography imaging is a gold standard in diagnosing the condition. The definitive treatment is surgery, recommended and encouraged for asymptomatic patients as well to reduce the risk of future complications. Whilst the approach to surgical management differs on a case-by-case basis, the main goal is to reduce the herniating organ and repair the defect. It is important to note that in severe cases, intestinal obstruction and strangulation may occur. We present a unique case of this very phenomenon in a patient diagnosed and treated as a case of strangulated Bochdalek hernia. We aim to highlight the importance of diagnosing this condition as clinical symptoms may be non-specific, and rapid surgical intervention is necessary.

## Introduction

Bochdalek hernia is a thoracoabdominal complex condition wherein there is an improper fusion of the posterolateral diaphragmatic foramina in utero during the ninth to tenth week of embryogenesis [1]. This results in herniation of abdominal contents into the thorax, constricting the developing lungs and potentially causing pulmonary hypoplasia. However, in asymptomatic patients, respiratory or abdominal symptoms may be experienced for the first time much later in life. However, the condition is rare in adults, with few cases reported in existing literature. When present, it can be severely life-threatening and prompts the need for quick surgical intervention [2]. The incidence has been previously reported to be 1 in 2200-12500 live births, with the left side being affected in 80-90% of all cases. In adults, it has been found that the colon is the most common organ to be affected in such cases as it traverses through the diaphragm and into the thorax, thereby increasing the risk of bowel obstruction [1]. 

## Case presentation

A 25-year-old previously healthy male presented to the emergency department with sudden onset epigastric and lower abdominal pain since the morning, associated with four episodes of non-bilious, non-bloody vomiting. The patient was traveling and was immediately brought from the airport to the hospital on account of his pain and vomiting. He denied any changes in stool consistency and reported opening his bowels the previous day and had been passing flatus without difficulty. 

**Figure 1:**
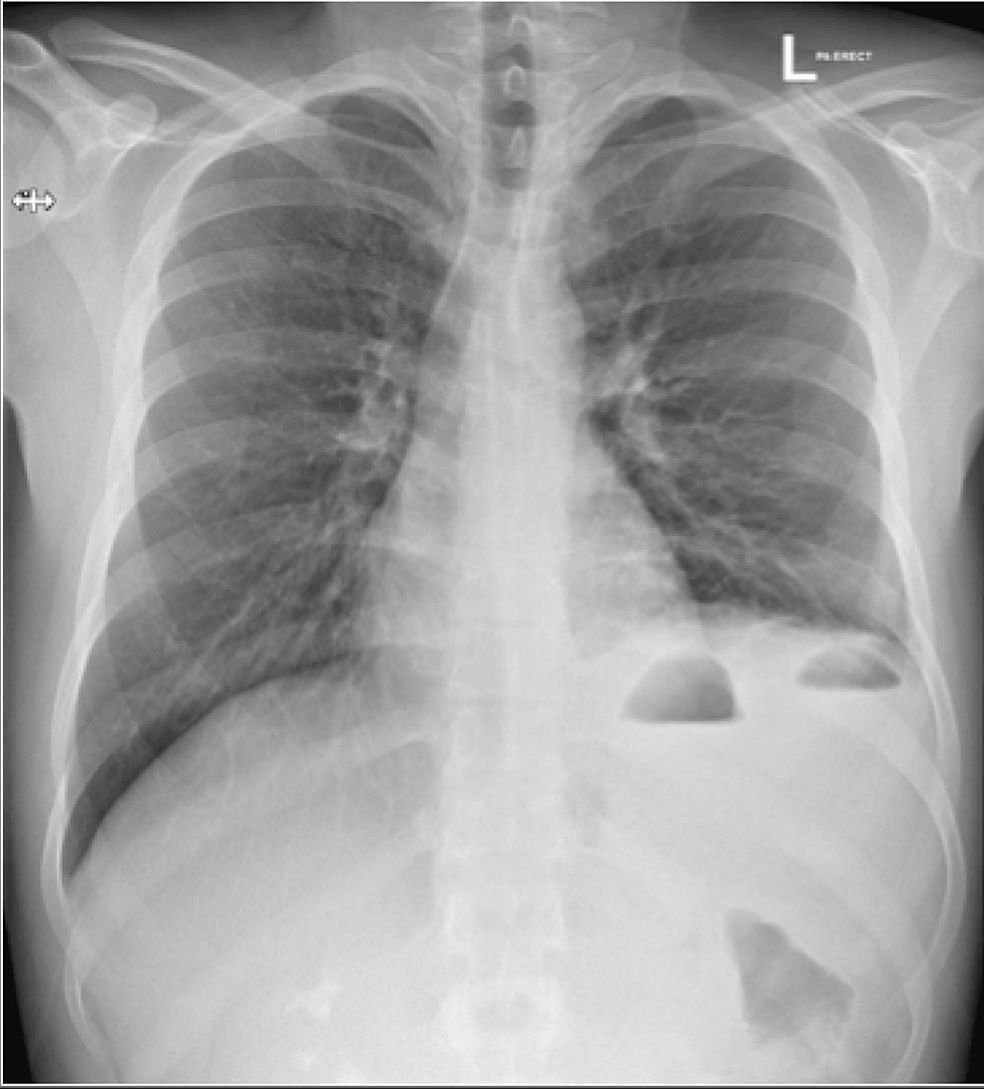
Chest X-ray showing an elevated left dome of the diaphragm with small air-fluid levels

He denied urinary complaints of urgency or dysuria. The patient denied any history of abdominal pain, recent trauma, or any previous hospital admission. He reported no significant past medical and surgical history. He denied any history of complications during his birth and reported normal childhood development. On examination, the patient was vitally stable. His abdomen was soft and lax, with mild tenderness in the central, epigastric, and lower abdomen without guarding or rigidity. Auscultation of the abdomen revealed decreased bowel sounds. Auscultation of the chest revealed no breath sounds in the left lower lobe of the lung. Laboratory investigations revealed leukocytosis with neutrophilic predominance, with all other parameters reported as normal. Chest radiograph revealed an elevated left dome of the diaphragm with basal atelectasis, and small air-fluid levels are seen beneath the left dome of the diaphragm (Figure 1). The right lung was clear with no abnormalities. 

**Figure 2:**
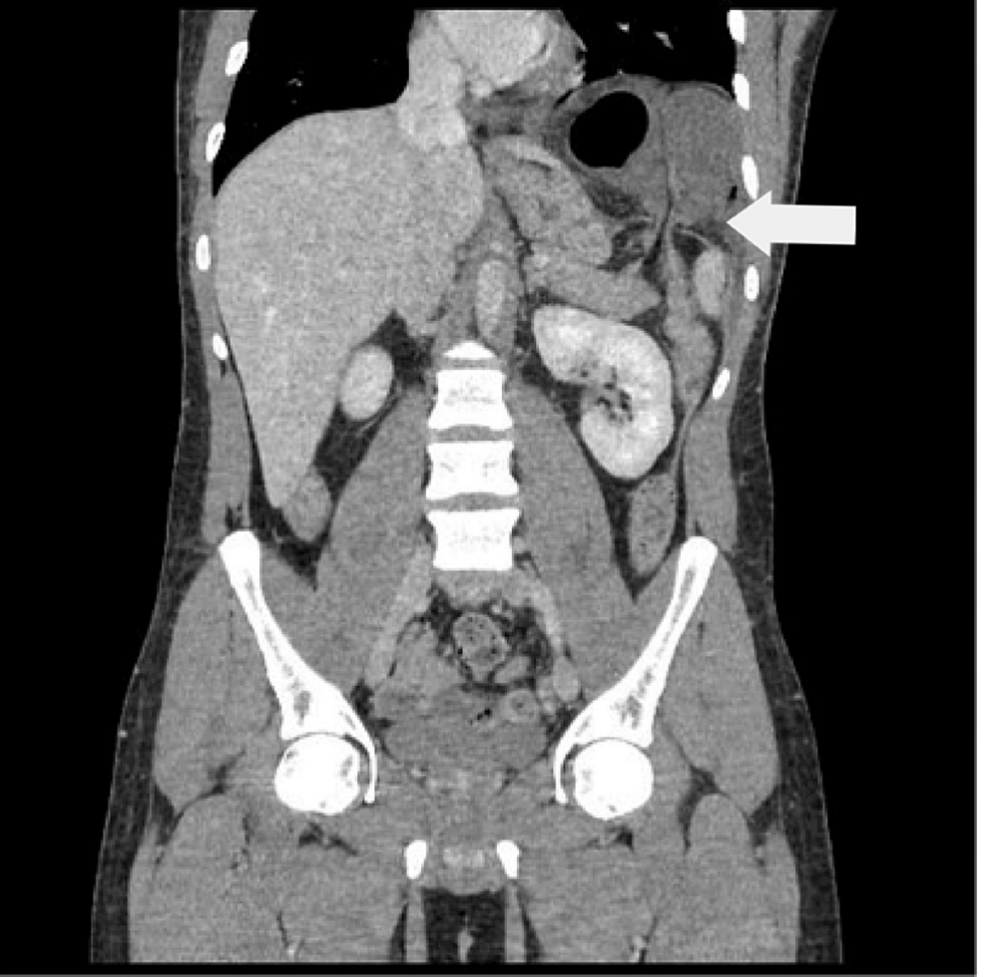
Computed tomography showing a left diaphragmatic hernia containing dilated small bowel loops with mesentery and omentum (white arrow)

Computed tomography (CT) of the abdomen with contrast was done, which confirmed a left diaphragmatic hernia due to a 1.9 cm-sized defect containing dilated small bowel loops with mesentery and omentum (Figure 2). The incarcerated bowel loop within the hernial sac shows significantly decreased wall enhancement and remarkable narrowing at both entry and exit of the incarcerated bowel loop, leading to significant dilatation, measuring up to 4.6 cm with air-fluid levels. Significant mesenteric fat stranding in the hernial sac is seen. The patient was diagnosed with a case of incarcerated and strangulated left diaphragmatic hernia and subsequently underwent an urgent diagnostic laparoscopy. 

**Figure 3:**
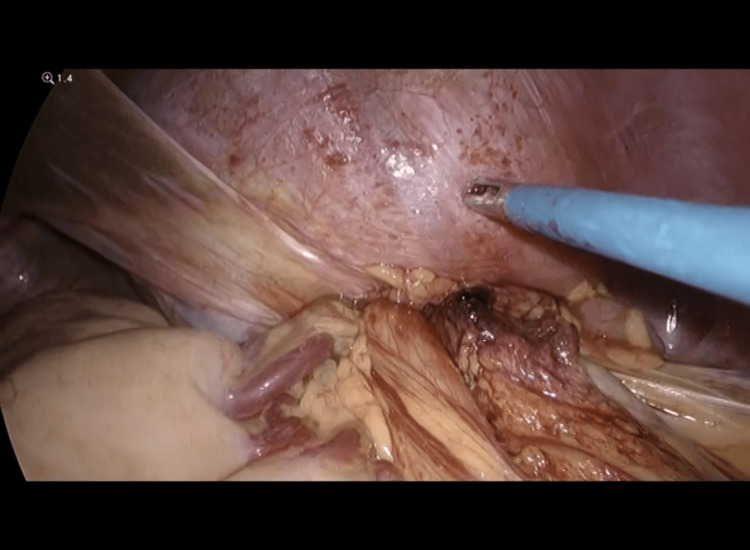
The strangulated segment of the bowel loops with omentum through the left diaphragmatic hernia defect

**Figure 4:**
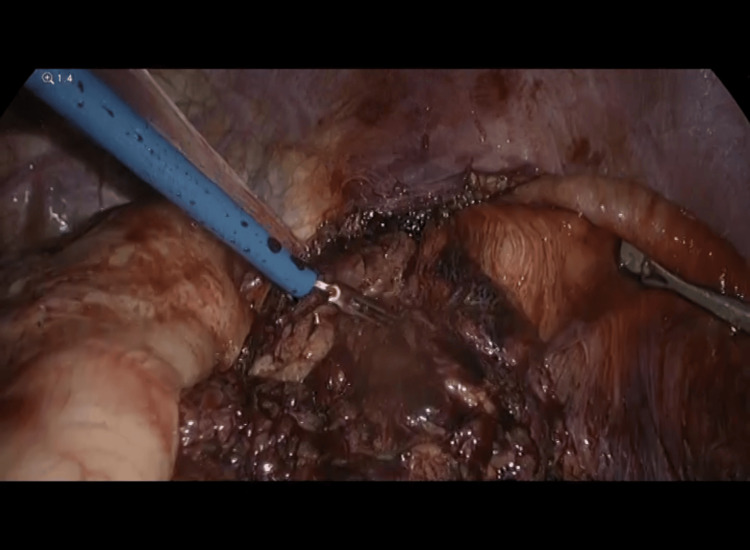
Releasing strangulated gangrenous loops of intestine and omentum after increasing the hernia defect

**Figure 5:**
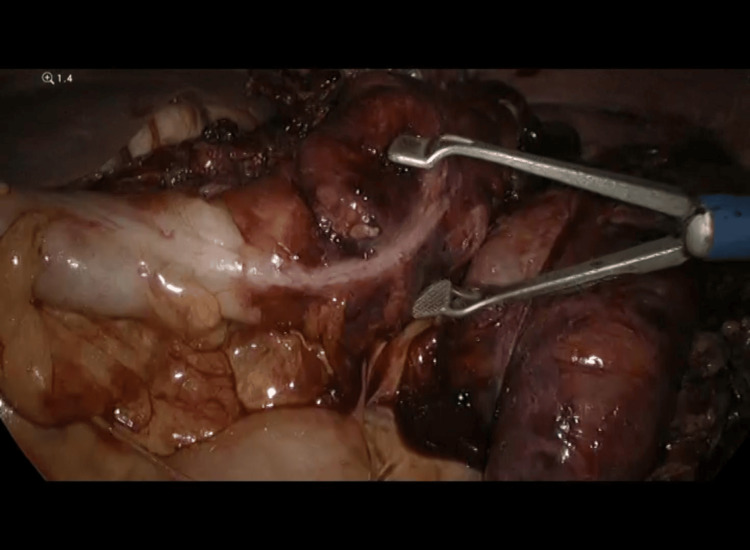
After being released from the hernia defect, the gangrenous distal transverse colon needed resection

Intra-operatively, a 3 cm x 2 cm defect was seen on the left posterior aspect of the diaphragm, containing a herniated ischemic segment of the large colon and ischemic omentum with surrounding adhesions at the neck of the hernia (Figure 3). Approximately 30 cm of the distal transverse colon, splenic flexure of colon, and proximal descending colon, along with greater omentum, was strangulated in the hernia defect (Figure 4 and Figure 5). Around 1.5 L of bloody fluid was present in the left hemithorax and was removed by suction evacuation. The stomach, small bowel, and other abdominal organs, including the spleen and liver, were unaffected. The hernia defect was extended laterally using Ligasure to facilitate the reduction of the ischemic colonic segment without perforation. 

**Figure 6:**
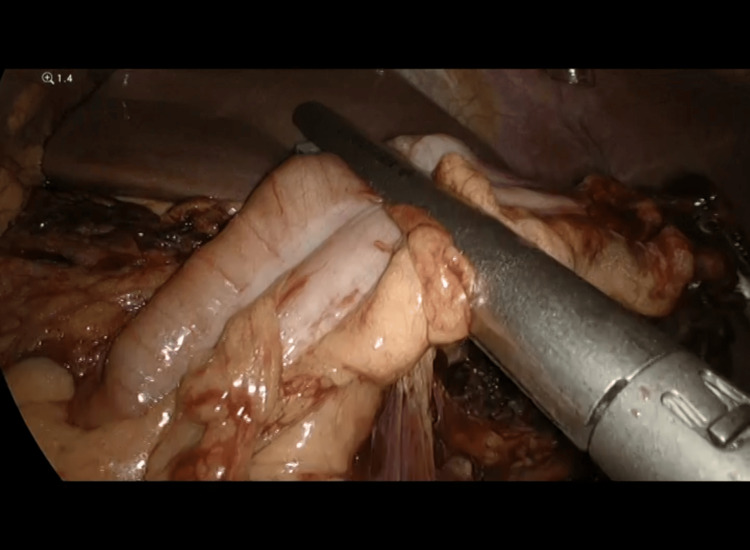
Laparoscopic resection of gangrenous distal transverse colon

**Figure 7:**
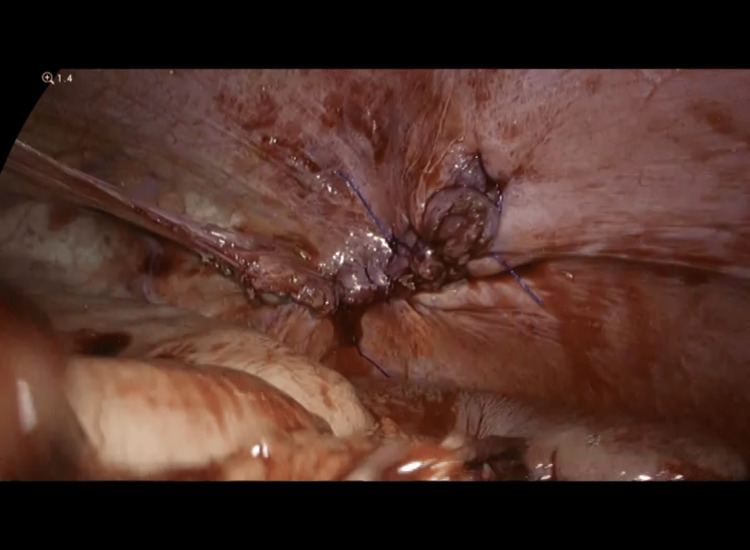
Repair of left diaphragmatic hernia defect without mesh

The herniated segment of the gangrenous colon with gangrenous greater omentum was resected (Figure 6). The hernia defect was repaired in two layers with non-absorbable sutures (Figure 7). No mesh was used to avoid mesh infection. Both proximal and distal colon loops were brought out together as a double barrel colostomy in the left upper abdomen. A left-side chest drain was inserted, and the patient was shifted to ICU. Post-operatively, the patient was recovering well without complications.

## Discussion

Etiology 

Genetic and environmental factors are the main predispositions to developing the condition, with mutations in more than 150 genes reported in existing literature [3]. Since these chromosomal aberrations are the leading causes of diaphragmatic hernias, karyotyping and prenatal imaging such as ultrasound or fetal magnetic resonance imaging (MRI) may be employed for early diagnosis and to improve patient outcomes and decrease mortality rates [4]. 

Whilst genetic factors are more influential, it has been evidenced that environmental factors such as deficiency of maternal vitamin A as well as pre-gestational maternal diabetes or hypertension have strong associations with the formation of diaphragmatic hernias [3,5]. 

Pathophysiology 

The diaphragm begins developing from the fourth to the twelfth week of gestation [6]. The Bochdalek foramen measures 2 cm x 3 cm and is found in the posterior aspect of the fetal diaphragm, superior to the suprarenal glands [7]. This structure facilitates communication between the pleural and peritoneal cavities through the pleuroperitoneal canal. During normal embryogenesis, closure of the foramen is expected between the ninth and tenth week, with the right canal closing earlier than the left. Bochdalek hernias result from the failure of fusion of the costal and crural aspects of the diaphragm. Since the left side closes later, there is a higher precedence of left-sided diaphragmatic hernias. The stomach, ileum, colon, and spleen are the intra-abdominal organs most commonly affected and herniate into the thorax [1]. In the rare instances of right-sided Bochdalek hernias, the liver, gallbladder, right kidney, and omentum are most likely to be affected [7,8]. 

Evaluating the patient 

In pediatric populations, respiratory symptoms, such as distress and cyanosis, predominate due to lung hypoplasia as a result of lung compression by the herniating abdominal contents. In such cases, physicians may be able to auscultate for bowel sounds in the chest. In adults, however, it may be an incidental finding in an asymptomatic patient [8]. It has been found that events that lead to increased intrathoracic pressure, such as exercise, childbirth, sexual intercourse, and the use of the Valsalva maneuver, may precipitate symptoms in previously unaffected patients [9]. Symptomatic patients may present with gastrointestinal complaints such as pain, pressure, dysphagia, and choking, as well as features of intestinal obstruction, as seen in our patient [1,8]. Dyspnea, frequent chest infections, and other pulmonary symptoms may be reported by patients, although they are less commonly experienced [7]. 

Diagnosing the condition is challenging for many physicians due to the vagueness of symptoms and the uncommonness of the condition. Although chest X-rays may be used as a first-line imaging modality to identify the presence of gas-filled bowels above the level of T10, as seen in our patient, they do not provide clear visualization of the herniated organ. Radionuclide imaging and abdominal ultrasounds, whilst helpful in exhibiting the discontinuity of the diaphragm, are far less superior compared to the gold-standard imaging technique of computed tomography (CT), which has a reported sensitivity of 78% for left-sided hernias and 50% for right-sided hernias [9]. 

Treatment 

It is recommended that all adult patients with Bochdalek hernias, regardless of symptom status, undergo surgery to rectify the defect and prevent complications in the future [10].

Bochdalek hernias may be surgically repaired depending on the presence of a strangulated bowel. In cases of intestinal strangulation, literature has previously reported similar outcomes irrespective of the transabdominal or transthoracic approach [11]. Whilst the surgical approach may vary, the end goal of the procedure is to effectively reduce the herniating organ and repair the diaphragmatic defect [12]. The use of prosthetic reinforcement like mesh has been largely debated. However, smaller defects can be reduced and reinforced with simple, non-absorbable sutures, as seen in our patient, who had a 1.9 cm-sized defect [13]. Patients with defects larger than 10 cm square may benefit from the use of mesh such as polypropylene, which has been shown to provide good structural support as well as improve tissue growth [13, 14]. Whilst there is limited evidence comparing biological versus synthetic mesh, both have been shown to lead to equally favorable patient outcomes [15]. In some cases where further fixation of the mesh is required, endo staplers have been proven to be quite useful [14]. 

## Conclusions

In adults, diaphragmatic hernias are termed Bochdalek hernias and may be found incidentally on imaging or through the development of symptoms. Intestinal obstruction is a serious life-threatening complication that could arise and warrants immediate surgical attention and intervention. The condition is often challenging to diagnose and treat due to the ambiguity of symptoms and the rarity of presentation. Gold-standard CT-guided imaging allows quick diagnosis and prompt management of the condition to overcome this life-threatening challenge.
